# Low alanine aminotransferase activity gene variant in a Siberian Husky with copper-associated hepatopathy

**DOI:** 10.1186/s12917-023-03681-6

**Published:** 2023-08-07

**Authors:** Christine Kim, John P. Loftus, Heather J. Huson

**Affiliations:** 1 IronHorse VetCare, 7660 Amador Valley Blvd E, Dublin, CA 94568 USA; 2 Department of Clinical Sciences, Cornell University College of Veterinary Medicine, 930 Campus Rd, Ithaca, NY 14853 USA; 3 Department of Animal Sciences, Cornell University College of Agriculture and Life Sciences, 201 Morrison Hall, 507 Tower Road, Ithaca, NY 14853 USA

**Keywords:** Copper-associated hepatopathy, Alanine aminotransferase, Chronic hepatopathy, Canine, Genetics

## Abstract

**Background:**

Alanine aminotransferase (ALT) is commonly used as a marker of hepatocellular injury. Increased serum ALT activity due to hepatocyte injury occurs in copper-associated hepatopathy (CuCH) and other necroinflammatory liver conditions. Blood ALT concentrations are frequently used to monitor therapy in cases of CuCH. Low serum ALT activities have been associated with an allele at a CFA13 locus.

**Case presentation:**

A 9-year-old female spayed Siberian Husky was diagnosed with CuCH (hepatic copper dry weight 2680 µg/g [normal, 120–400 µg/g; toxic, > 1500 µg/g]) and a normal ALT (78 U/L; reference range, 10–125 U/L). Mild hepatocellular necrosis was evident histologically. Genetic testing (Embark) revealed that the dog was heterozygous for the low ALT activity gene allele.

**Conclusions:**

This case report illustrates the clinical implications for diagnosing and managing necroinflammatory liver disease such as CuCH in dogs with a low ALT activity genotype.

## Background

Serum alanine aminotransferase (ALT) activity is commonly used as a biomarker for hepatocellular injury. In dogs, ALT is highly specific for hepatocellular necrosis and is present predominantly in the cytosol [1, 2]. Various hepatic necroinflammatory conditions can lead to hepatocellular necrosis, including infectious, neoplastic, or chronic inflammatory diseases.

Copper-associated hepatopathy (CuCH) is the most common toxic-injury-induced chronic hepatopathy in dogs [3, 4]. Contributing factors include genetic predisposition and dietary copper levels. Predisposed breeds include the Bedlington Terrier, Dalmatian, Labrador Retriever, Doberman Pinscher, and West Highland White Terrier, but CuCH can occur in any breed. The genetic basis of CuCH in two breeds is described and associated with cellular copper trafficking mechanisms. The Bedlington Terrier was the first breed where CuCH was extensively studied, and a *COMMD1* (*MURR1*) mutation is responsible for CuCH in this breed [5, 6]. More recently, CuCH has been increasingly recognized in the Labrador Retriever, where variants in the *ATP7B* and *ATP7A* genes have been implicated as a predisposing or protective factor, respectively, for this breed’s CuCH [7]. Hepatic copper accumulation (typically greater than 1000 µg/g liver dry weight [dw]) induces oxidant injury, manifested by histological morphologic changes or increased serum ALT activity [4].

Lower serum ALT activity has been associated with an allele (A) at a CFA13 locus in dogs of many breeds, although allele causality has not been proven [8]. In healthy dogs, homozygous AA and heterozygous AG dogs had lower ALT activities than dogs homozygous for the GG allele. Dogs that were AA homozygous with liver disease or injury had the lowest ALT activities, often in the reference range. In a recent study evaluating genetic traits in northern working breeds, the most common health trait identified by a commercial genetic testing panel in Alaskan Sled Dogs and Siberian Huskies was lower ALT activity [9]. We present a case of a Siberian Husky with the low ALT activity trait on Embark testing with severe hepatic copper accumulation and histologically documented hepatocellular necrosis.

## Case presentation

A 9-year-old female spayed Siberian Husky weighing 32.1 kg was presented to an emergency service at a veterinary specialty hospital for a decreased appetite of a few days duration in January of 2022. The dog’s only previously diagnosed active medical problem was hypothyroidism, diagnosed one year prior. The dog’s only medical treatments at presentation included levothyroxine (0.6 mg orally twice daily). She was being fed a Kirkland brand adult dry dog food. Physical examination revealed no abnormalities. During this initial evaluation, blood was drawn for a complete blood count, which did not reveal any abnormalities. A serum chemistry panel (Table 1) showed an elevated alkaline phosphatase (ALP) activity (438 U/L; reference range, 23–212 U/L) and was otherwise normal (ALT 78 U/L; reference range, 10–125 U/L).

**Table 1:** Summary of salient serum biochemistry values (Idexx Reference Laboratory)

Parameter	Results (initial presentation, 1/22/22)	Results (follow-up, 4/22/22)	Results (follow-up, 10/22/22)	Reference Range
Albumin (g/L)	36	33	30	27–39
ALP (U/L)	438	424	371	5-160
ALT (U/L)	78	89	43	18–121
AST (U/L)	NR	34	23	16–55
Bilirubin, total (mcmol/L)	NR	1.71	1.71	0.0-5.1
Cholesterol (mmol/L)	6.6	5.6	5.5	3.4-9.0
GGT (U/L)	NR	2	1	0–13
Globulin (g/L)	46	34	33	24–40
Glucose (mmol/L)	7.4	4.7	6.2	3.5–6.3
Protein, total (g/L)	NR	67	63	55–75
Urea nitrogen (mmol/L)	NR	5.0	5.4	3.2–11.1

*ALP = alkaline phosphatase, ALT = alanine aminotransferase, AST = aspartate aminotransferase, NR = not reported*

Thoracic radiographs were unremarkable. Abdominal ultrasonography revealed an 11 × 8 cm mixed echogenic mass originating from the body of the spleen. The mass was expansile and associated with hyperechoic surrounding mesentery, raising concern for a recent bleeding event. The liver size was at the lower end of normal, with a homogenously isoechoic parenchyma.

**Fig. 1:**
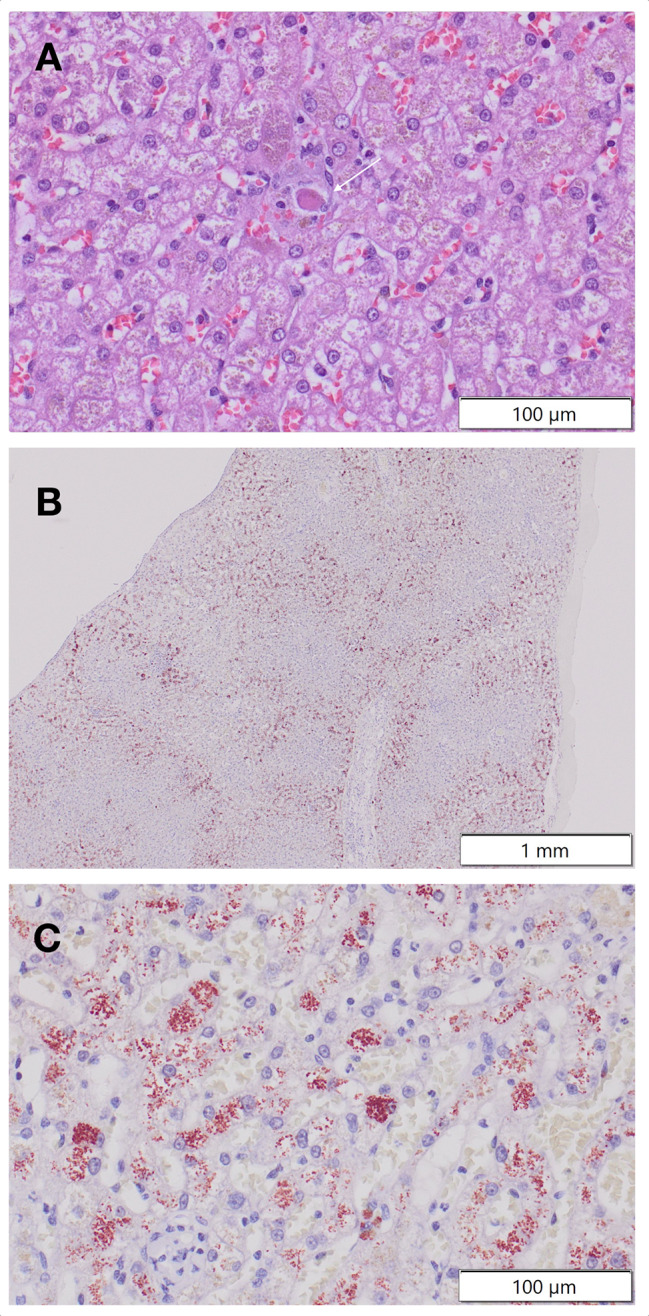
**Representative photomicrographs of the dog’s liver biopsy sample.** (A) High-power image of a hematoxylin and eosin stained histologic section of the patient’s liver, demonstrating a necrotic cell with hypereosinophilic cytoplasm (arrow). Note cytoplasmic pigment in the necrotic and adjacent hepatocytes. (B & C) Rhodanine staining at low (B) and high (C) power confirmed the pigment represented copper

A splenectomy and liver biopsies were performed the following day. Histopathology of the spleen was consistent with nodular lymphoid hyperplasia with congestion, hemorrhage, and occasional necrosis. Histologic evaluation of the liver revealed mild, diffuse vacuolar hepatopathy and occasional midzonal individual hepatocellular necrosis (Fig. 1A) with moderate multifocal nodular histiocytic and less lymphocytic and neutrophilic infiltrates. Minimal multifocal lymphoplasmacytic portal hepatitis was present with mild portal fibrosis and bile duct hyperplasia. Marked subjective centrilobular to midzonal hepatocellular copper accumulation was suspected on hematoxylin and eosin stained sections. Rhodanine staining revealed large numbers of centrilobular to midzonal hepatocytes with moderate to frequent large amounts of cytoplasmic copper (Fig. 1B&C). Quantitative copper testing (Colorado State University) confirmed a markedly elevated hepatic copper concentration of 2680 µg/g dw (normal, 120–400 µg/g dw; toxic, > 1500 µg/g dw).

The dog was presented to the Internal Medicine Service at IronHorse VetCare for further evaluation of a copper-associated hepatopathy in April 2022. A serum chemistry panel (Table 1) was re-evaluated, revealing a persistently elevated ALP (424 U/L; reference range, 5-160 U/L) and a normal ALT (89; reference range, 18–121 U/L). Paired serum bile acid concentrations were within normal limits. The dog was transitioned to a low copper (approximately 1 mg/1000 kcal) veterinary therapeutic hepatic diet with ~ 2 tablespoons of cottage cheese or chicken breast per cup or can of diet provided for additional protein. D-penicillamine treatment was prescribed at 300 mg (10 mg/kg) by mouth once daily for 14 days, then increased in frequency to twice daily.

The dog’s normal ALT activity in the face of severe copper accumulation and hepatocellular necrosis prompted genetic screening through a commercial laboratory that tests for the A allele previously described [8] as associated with low ALT activity (Breed + Health Test, Embark Veterinary, Inc., 320 Summer Street, Floor 6, Boston, MA 02210, https://embarkvet.com/). Results disclosed that the dog had one copy of the low ALT activity trait variant, indicating she was heterozygous AG.

Six months (October 2022) after starting D-penicillamine, the dog was re-evaluated at another veterinary hospital. The dog’s ALT remained normal (43 U/L, reference range 18–121 U/L), although it was now approximately half of the previously measured values (Table 1). The ALP was persistently elevated (371, reference range 5-160 U/L). The dog’s owner reported that she remains clinically well with a normal appetite and energy level. Penicillamine was discontinued, and a low copper diet was recommended to be continued indefinitely.

## Discussion and conclusions

This case report illustrates the clinical impact of low ALT activity gene variants in dogs and the interpretation of routine biochemical testing. Patients with this trait may be underdiagnosed or diagnosed later in the course of necroinflammatory hepatic conditions. Additionally, monitoring ALT in these cases may not be as suitable a surrogate biomarker for hepatocellular injury as it is in dogs with normal ALT activity genotypes.

Evidence of hepatic damage in CuCH typically manifests as increased serum ALT activity, histologic changes, or both when hepatic copper concentrations exceed 1000 µg/g dw and almost always occurs at concentrations over 1500 µg/g dw. In this case, ALT concentrations were normal despite the histologic presence of multifocal inflammation and necrotic hepatocytes. At least two possibilities for this observation exist. First, this degree of necroinflammatory disease was insufficient to result in increased serum ALT activity in this dog and unrelated to the CFA13 locus genotype. The second possibility is that this dog’s genotype contributed to its discordantly normal serum ALT activity. The normal serum AST activities measured in this case may support the former assertion. However, in one study, the median AST:ALT ratios in dogs with hepatic fibrosis and necroinflammatory changes were approximately 0.25 [10]. Thus, dogs with mild ALT increases would frequently have normal serum AST activities. The original study documenting the low ALT activity gene variant did not include serum AST in their model. In the primary author’s dissertation defense, AST was analyzed, and they found the opposite trend compared to ALT between AST activities and the A allele at the CFA13 locus. Interestingly, however, many heterozygous dogs with hepatic disease had AST activities in the normal range [11]. It is impossible to irrefutably explain this patient’s normal ALT activity by its genotype alone. However, given the histologic findings and the reduction in ALT after treatment, we suspect the dogs’ low ALT activity genotype is the most likely explanation for this patient’s discordant clinical pathology results and liver histologic lesions.

Discordance and variability between histologic disease severity, serum hepatic enzyme activities, or hepatic copper concentrations have been documented previously in canine liver diseases [3, 10, 12–14]. Chronic hepatitis can be present in dogs with normal serum liver enzyme activities, despite being the most common biochemical abnormality in dogs with chronic hepatitis [4]. In two studies including dogs with CuCH, ALT activity was increased in all cases where it was measured; however, hepatic copper concentrations were not measured, precluding more direct comparisons [15, 16]. This case represents an additional challenge as serum ALT activity is frequently used as a surrogate marker for hepatocellular injury to guide therapy in dogs with CuCH [4]. Serum concentrations of some microRNAs, such as miR-122, are increased in dogs with hepatocellular injury [17–20]. MicroRNA biomarkers may be helpful in screening and monitoring cases of chronic hepatitis with low ALT activity genotypes but, unfortunately, are not available for routine diagnostic testing. This dog’s increased ALP activity aligns with the histologically observed vacuolar changes and may be partly reactive to CuCH. Therefore, monitoring ALP activity may provide some metric of treatment response, although it may be less specific for copper chelation efficacy than ALT. Ultimately, liver biopsy is the gold standard measure of copper chelation efficacy. However, treatment success is often gauged by ALT monitoring. Thus, there may be a greater need to consider post-treatment liver biopsy to monitor chelation efficacy in dogs with low ALT activity genotypes.

This case illustrates the potential impact of genotype on the interpretation of serum ALT activity in dogs with necroinflammatory hepatic diseases. Genetic testing may guide diagnostic and monitoring decisions that frequently rely on ALT as a surrogate marker of hepatocellular injury. Further investigation of ALT allele variants in dogs with necroinflammatory hepatic disease may help explain some of the discordance observed between the histologic severity of hepatocellular injury and serum ALT activities. Prospective studies are needed to understand better the impact of low ALT genotypes on serum biochemical screening and monitoring for dogs with hepatic necroinflammatory disease. Until more studies are conducted, screening for this genotype may be considered in all dogs with discordant hepatic histologic and biochemical results. This case also illustrates the need for additional clinically available biomarkers for hepatic necroinflammatory conditions, such as CuCH in the dog.

## Data Availability

The patient data used and/or analyzed during the current study are available in anonymized format from the corresponding author upon reasonable request.

## List of abbreviations

ALP: alkaline phosphatase
ALT: alanine aminotransferase
AST: aspartate aminotransferase
CuCH: copper-associated chronic hepatopathy
dw: dry weight
NR: not reported
